# Angular photogrammetric analysis of the soft tissue profile in 12-year-old southern Chinese

**DOI:** 10.1186/s13005-014-0056-3

**Published:** 2014-12-24

**Authors:** Cindi SY Leung, Yanqi Yang, Ricky WK Wong, Urban Hägg, John Lo, Colman McGrath

**Affiliations:** Paediatric Dentistry and Orthodontics, Faculty of Dentistry, The University of Hong Kong, Hong Kong, HKSAR, China; Department of Dentistry Maxillofacial Surgery, United Christian Hospital, Hong Kong, HKSAR, China; Oral and Maxillofacial surgery, Faculty of Dentistry, The University of Hong Kong, Hong Kong, HKSAR, China; Periodontology and Public Health, Faculty of Dentistry, The University of Hong Kong; Prince Philip Dental Hospital, 34 Hospital Road, Hong Kong, SAR China

**Keywords:** Photogrammetric analysis, Soft tissue profile, 12-year-old southern Chinese

## Abstract

**Introduction:**

To quantify average angular measurements that define the soft tissue profiles of 12-year-old southern Chinese and to determine gender differences.

**Materials and methods:**

A random population sample of 514 12-year-old children was recruited (about 10% of a Hong Kong Chinese birth cohort). Photographs were taken in natural head posture and 12 soft tissue landmarks were located on the photos to measure 12 angular measurements using ImageJ (V1.45s) for Windows. Approximately 10% of photographs were reanalyzed and method error was calculated. Angular norm values for the 12 parameters were determined and gender differences were assessed using 2 sample *T*-test with 95% confidence interval.

**Results:**

The response rate was 54.1% (278/514). Norm values for the 12 angular measurements were generated. The greatest variability was found for the nasolabial (Cm-Sn-Ls) and labiomental (Li-Sm-Pg) angles. Gender differences were found in 4 angular parameters: vertical nasal angle (N-Prn/TV) (p < 0.05), cervicomental angle (G-Pg/C-Me) (p < 0.001), facial convexity angle (G-Sn-Pg) (p < 0.01) and total facial convexity angle (G-Prn-Pg)(p < 0.01).

**Conclusion:**

Norm values for 12 angular measurements among 12-year-old southern Chinese children were provided and some variability noted. Gender differences were apparent in several angular measurements. This study has implications in developing norm values for southern Chinese and for comparison with other ethnic groups.

## Introduction

The assessment of the patients’ soft tissue profile is a critical step in orthodontic diagnosis and treatment planning. Achieving a pleasing esthetic profile is an important goal of orthodontic therapy, and can influence the treatment decision to extract teeth or not [1].

In the early twentieth century, orthodontic treatment goals were limited to achieving a functional occlusion and stability of the dentition. Although soft tissue esthetics were noted, little could be done to alter the soft tissue profile [2].

The introduction of orthopaedic and craniofacial surgical techniques in the 1960s and 1970s has allowed facial harmony to be incorporated into treatment goals [3]. The introduction of skeletal anchorage techniques has also produced positive results. These advances in technology have allowed greater improvements in facial profile aesthetic outcomes from orthodontic care.

In the past, orthodontic diagnosis and treatment planning has relied primarily on data from cephalometric studies. Numerous approaches have been developed for cephalometric analysis and many included soft tissue assessments [4-8]. Although cephalometric assessment serves as a useful guideline towards diagnosis and treatment planning, soft tissue profiles are of important consideration to attain improved facial appearance.

In cephalograms, the soft tissue structures are recorded only in profile and limited to the anterior-most outline. Furthermore, patients are typically unaccustomed to viewing and interpreting cephalograms or their tracings whereas photographs, on the other hand, provide a more amenable approach to documenting and assessing the soft tissues of the face [9].

There is marked variation in the soft tissue covering the dentoskeletal framework [10,11]. Longitudinal studies have indicated that the soft tissue profile does not directly follow the underlying skeletal profile [12]. Thus there is a need to ‘directly’ study the parameters of the soft tissue lateral profile. Various authors have studied the soft tissue profiles using photogrammetry [13-16].

An increase in dental awareness has created a great demand for orthodontic treatment in Chinese populations [17]. Whilst norms have been established for Caucasian populations regarding both cephalometric readings and lateral soft tissue profile parameters, to date, there is a lack of study in this area for the Chinese population. There is thus, a great need to establish Chinese population norms.

The aims of the present study were (1) to quantify average parameters that define the soft tissue profiles’ of 12-year-old southern Chinese; and (2) to describe any gender differences found.

## Materials and methods

### Sample

A population-wide epidemiological study was conducted among 12-year-old children in Hong Kong SAR, China. Ethical approval was obtained by the local Institutional Review Board (Reference Number: UW 09-453). Support was obtained by a grant from the Research Grants Council of the Hong Kong Special Administrative Region, China. A random sample of 10% of all secondary schools in Hong Kong SAR was selected and within each school children were invited to participate. Their parents provided written informed consent and the children provided their ascent. A sample of 514 (259 males, 255 females) was recruited, approximately 10% of a Chinese birth cohort. Sixteen subjects (3.1%) who had history of orthodontic treatment or were undergoing active orthodontic treatment were excluded from the final sample.

### Data collection

#### Photographic set up and record taking

Data collection was done ‘in the field’ (non-clinical setting) at available classrooms designated by each school. The photographic set up consisted of a Canon EOS 400D (Canon, Shimomaruko, Ohta-ku, Tokyo, Japan) camera with Canon EF-S 60mm f/2.8 Macro USM Lens and Canon MR-14EX TTL Macro Ring lite Flash. Subjects were positioned against a scale backdrop of 1cm increment with plumbline which indicated the true vertical (TV). A vertical standing mirror was positioned out of the frame perpendicular to the left side of the background set up for improved reproducibility of the natural head posture [18].

Subjects were asked to remove glasses or other accessories, which may obstruct the profile. They were then instructed to a standing position, asked to relax with both arms hanging down at their sides and look straight into their eyes in the mirror with lips in a relaxed position. The right side profile was taken in natural head posture (NHP).

#### Selection of landmarks and angular parameters

**Figure 1:**
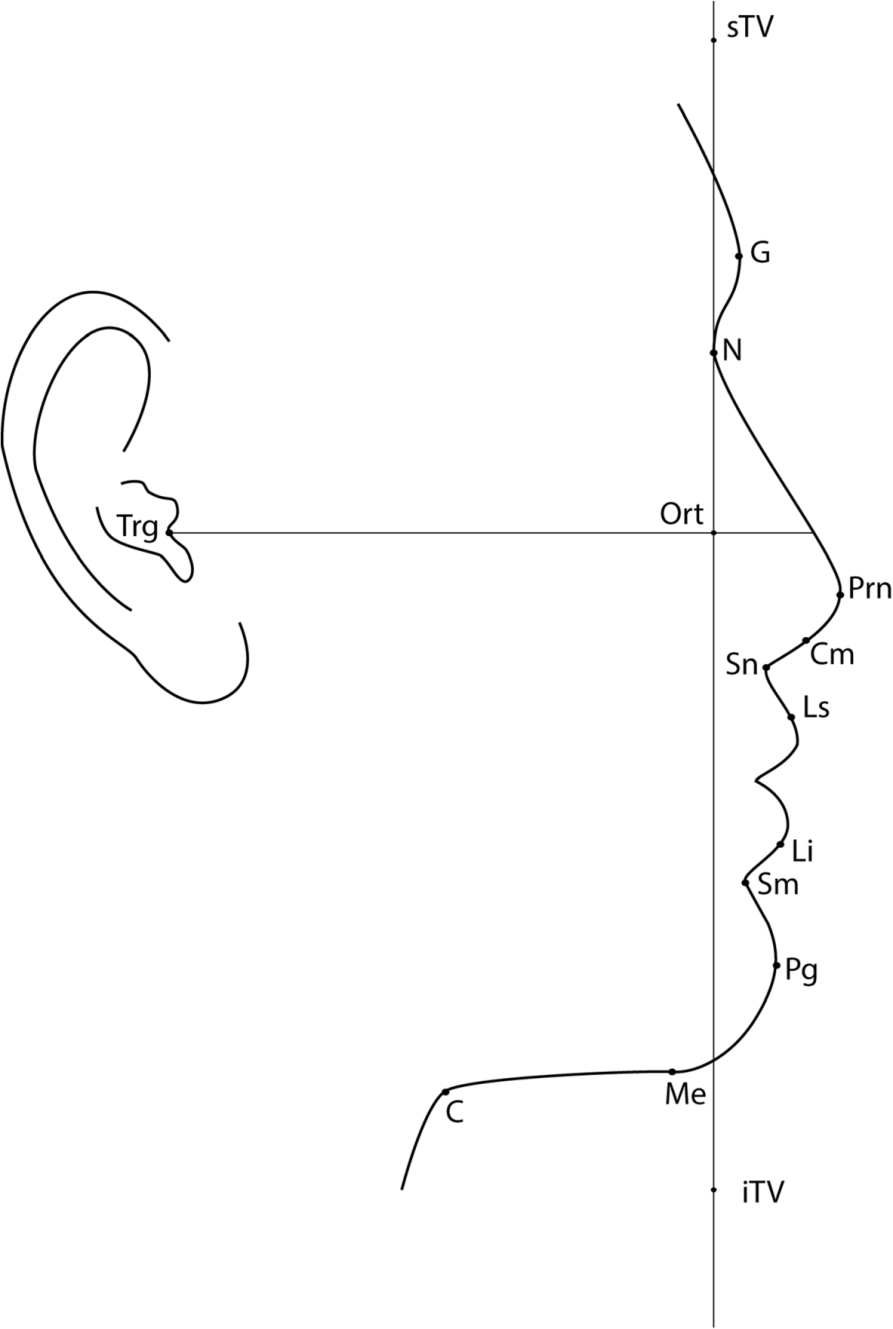
**Soft tissue landmarks and reference lines.** G, glabella; N, nasion; Prn, pronsal; Cm, columella; Sn, subnasal; Ls, Labiale Superius, Li, Labiale Inferius; Sm, supramental; Pg, pogonion; Me, menton; C, cervical; Trg, tragus; sTV, superior point of true vertical; iTV, inferior point of true vertical; Ort point, junction point of true vertical and true horizontal. Reference lines: sTV-iTV, true vertical; N-Ort (parallel to TV through nasion), true vertical in nasion; Trg-Ort (perpendicular to TV through Trg), true horizontal.

**Figure 2:**
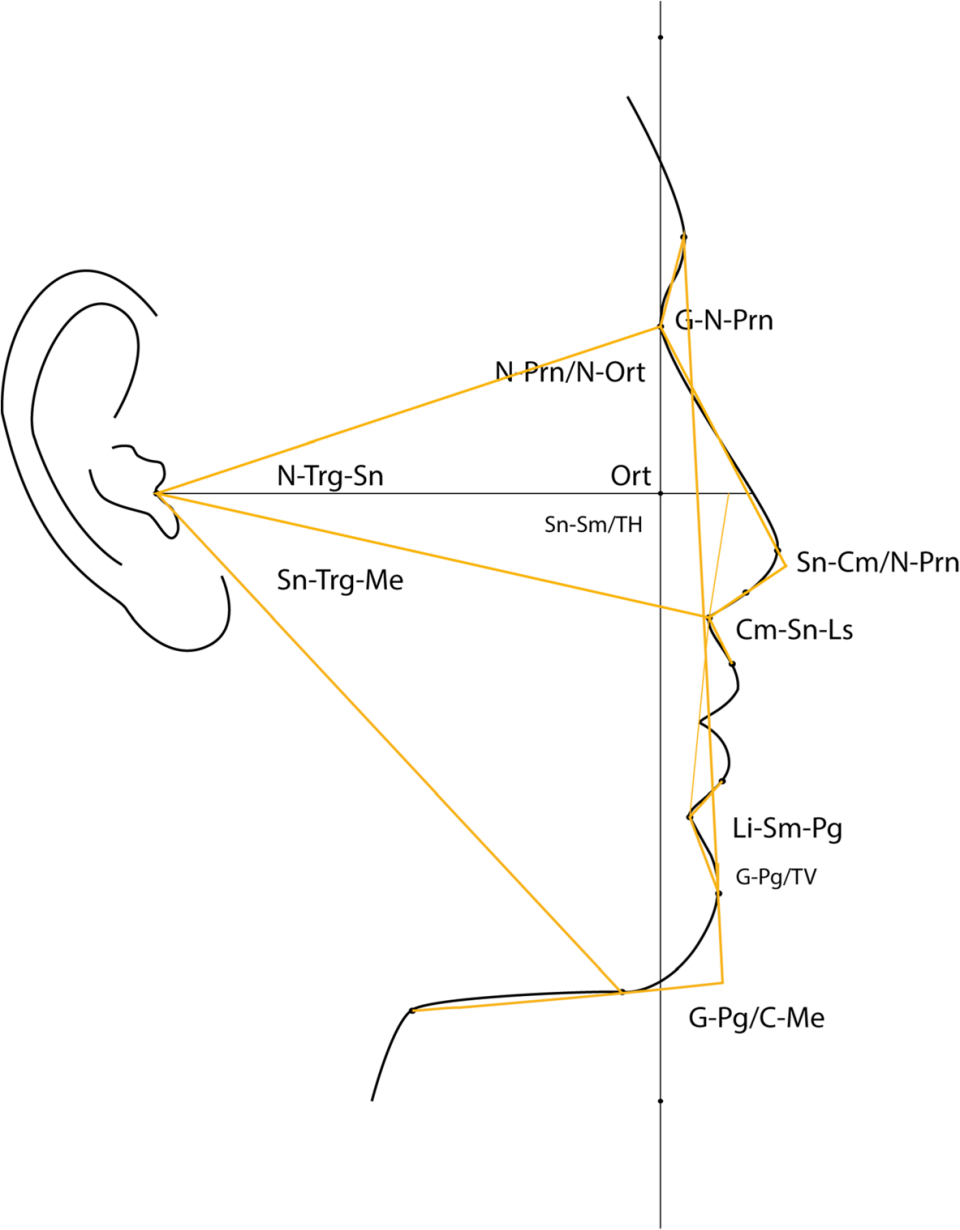
**Angular measurements: G-N-Prn, Nasofrontal angle; N-Prn/N-Ort, vertical nasal angle; Cm-Sn-Ls, nasolabial angle; Li-Sm-Pg, labiomental angle; Sn-Cm/N-Prn, Nasal angle; G-Pg/C-Me, cervicomental angle; N-Trg-Sn, angle of medium facial third; Sn-Trg-Me, angle of inferior facial third; Sn-Sm/TH, angle of head position; G-Pg/TV, glabella pogonion vertical.**

**Figure 3:**
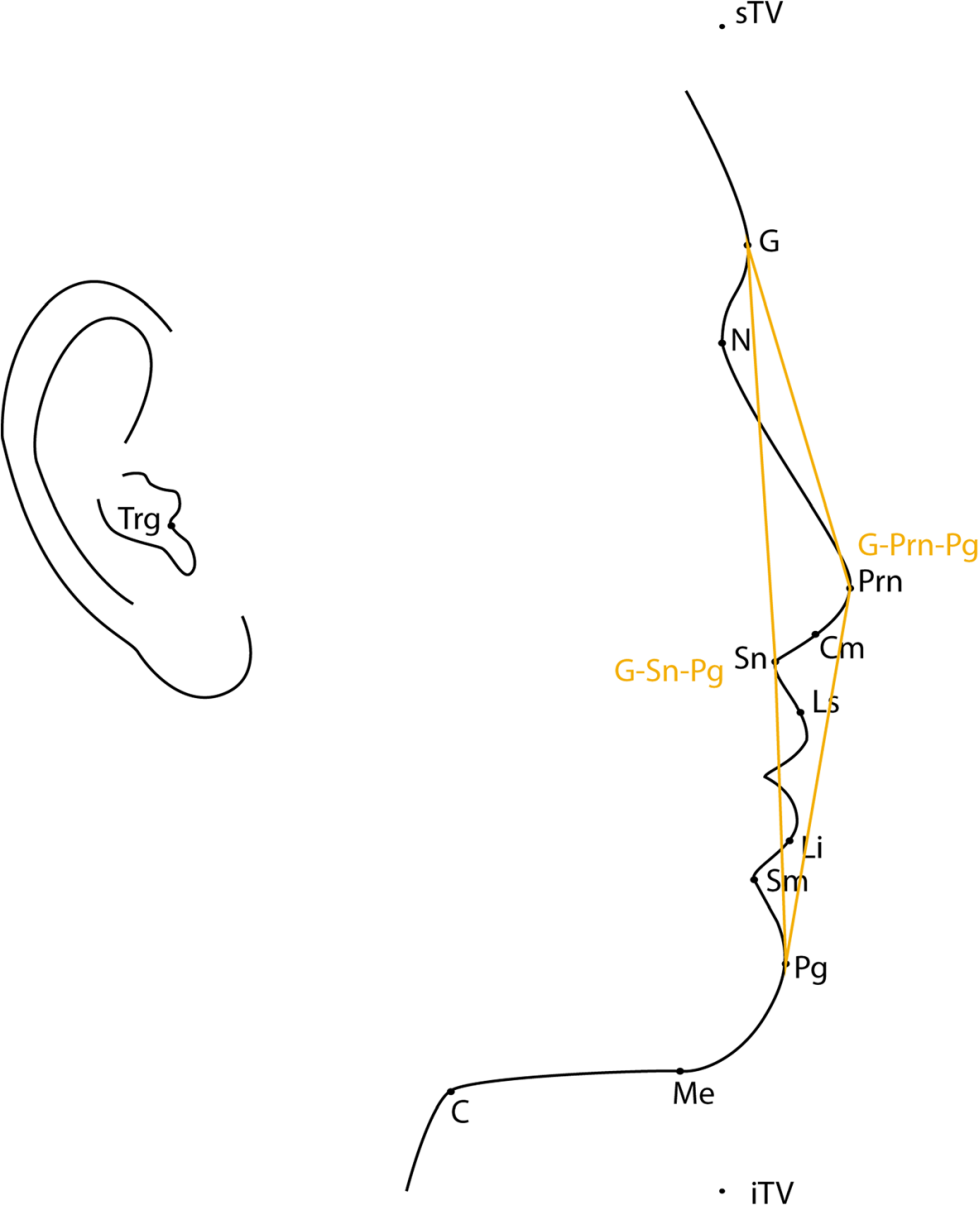
**Angular measurements: G-Sn-Pg, angle of facial convexity; G-Prn-Pg, angle of total facial convexity.**

Twelve soft tissue landmarks (Table 1, Figure 1) and 12 angular parameters (Table 2, Figures 2 and 3) were selected based on a modification of Fernández-Riveiro [19] and Malkoç [20] photogrammetric methods and in addition, a variable (G-Pg/TV) to assess the angle of head posture was also assessed.

**Table 1:** **Landmarks, abbreviations and reference lines employed in this study**

**Abbreviations**	**Landmark/reference point**
**G**	Glabella
**N**	Nasion
**Prn**	Pronasal
**Cm**	Columella
**Sn**	Subnasale
**Ls**	Labiale Superius
**Li**	Labiale Inferius
**Sm**	Supramental
**Pg**	Pogonion
**Me**	Menton
**C**	Cervical
**Trg**	Tragus
**sTV**	Superior point of true vertical
**iTV**	Inferior point of true vertical
**Ort**	Junction point of true vertical & true horizontal
**Reference point and lines**	
**True vertical**	sTV – iTV
**True vertical in nasion**	N-Ort (parallel to TV through Nasion)
**True horizontal**	Trg-Ort (perpendicular to TV through Trg)

**Table 2:** **Description of angular measurements**

**Parameter**	**Description**
**Nasofrontal**	G-N-Prn
**Vertical nasal**	N-Prn/TV
**Nasolabial**	Cm-Sn-Ls
**Labiomental**	Li-Sm-Pg
**Nasal angle**	Sn-Cm/N-Prn
**Cervicomental**	G-Pg/C-Me
**Medium facial third**	N-Trg-Sn
**Inferior facial third**	Sn-Trg-Me
**Head position**	Sn-Sm/TH
**Facial convexity**	G-Sn-Pg
**Total facial convexity**	G-Prn-Pg
**Glabella pogonion vertical**	G-Pg/TV

#### Selection of photographs

Photographs were screened for ‘usability’ i.e. feasibility of identifying landmarks and angular measurements. Photographs were excluded if: 1) landmarks were obstructed or unclear; 2) ‘out of focus’; 3) subjects exhibiting obvious lip muscle strain or open mouth posture; 4) subjects who exhibited facial expressions that were not neutral (i.e smiling); 5) head tilting up or down; 6) Photographs where the plumbline or scale backdrop were not visible.

A final sample of 278 subjects (166 males, 112 females) was recruited.

#### Digitalization

Photographs were orientated to true vertical using ImageJ V1.45s and cropped to show the head and neck area only. The 12 soft tissue landmarks were located (Table 1, Figure 1) to measure 12 angular measurements (Table 2, Figure 2, 3). Two arbitrary points were defined parallel to the true vertical through the N point to identify the ‘true vertical in Nasion’. Reference lines were created using Microsoft Paint®. The same trained and calibrated examiner conducted all photographic measurements.

#### Statistical analysis

Descriptive statistics were produced of mean, standard deviation and range (maximum, minimum) for each parameter. Gender differences were determined using t- test statistics with 95% confidence interval provided by SPSS (V19) (IBM Corporation, Armonk, New York, USA).

To assess method error, about 10% of randomly selected subjects were re-analyzed for intra-examiner method error analysis as calculated by Dahlberg’s formula [21]. Intra-class correlation coefficients were calculated by SPSS (V19).

## Results

Among the 514 subjects, 45.9% (236) were excluded in the selection of photographs according to the exclusion criteria, providing an overall response rate of 54.1% (278/514).

Intra-examiner method error was determined based on about 10% of the sample (Table 3). The angles with the highest method error were the Nasolabial: 2.50°, labiomental angle: 2.76° and nasal angle: 2.52°. ICC values (Table 4) were greater than 0.8 for all angles, indicating good reliability.

**Table 3:** **Method error according to Dahlberg’s formula**

**Parameter**	**Method error (°)**
**Nasofrontal**	0.76
**Vertical Nasal**	0.3
**Nasolabial**	2.5
**Labiomental**	2.76
**Nasal angle**	2.52
**Cervicomental**	0.5
**Medium facial third**	0.28
**Inferior facial third**	0.38
**Angle of head position**	0.25
**Facial convexity**	0.44
**Total facial convexity**	0.45
**Glabella-pogonion vertical**	0.11

**Table 4:** **Intra-class correlation coefficients**

**Parameter**	**ICC value**
**Nasofrontal**	0.982
**Vertical Nasal**	0.991
**Nasolabial**	0.945
**Labiomental**	0.939
**Nasal angle**	0.89
**Cervicomental**	0.997
**Medium facial third**	0.982
**Inferior facial third**	0.975
**Angle of head position**	0.998
**Facial convexity**	0.993
**Total facial convexity**	0.993
**Glabella-pogonion vertical**	0.998

Descriptive statistics (Mean (SD) and range) of the 12 angular parameters are provided in Table 5. The greatest variability was found for the nasolabial (male: 64.56° to 132.80°, female: 73.33° to 123.89°) and labiomental (male: 93.36° to 158.11°, female: 98.27° to 164.11°) angles. These angles also exhibited the highest method error.

**Table 5:** **Descriptive data for measurements in males and females**

	**Male (n = 166)**				**Female (n = 112)**				
**Parameter**	**Mean**	**S.D**	**Min**	**Max**	**Mean**	**S.D**	**Min**	**Max**	**P value**
**Nasofrontal**	143.94	4.97	126.52	157.36	144.68	4.51	131.17	157.14	0.212
**Vertical Nasal**	26.95	3.69	18.25	39.34	25.97	3.67	16.84	34.80	0.031*
**Nasolabial**	99.03	11.52	64.56	132.80	99.05	10.24	73.33	123.89	0.991
**Labiomental**	132.56	12.46	93.36	158.11	135.30	11.43	98.27	164.11	0.064
**Nasal angle**	91.77	8.74	66.18	110.68	91.18	7.87	67.59	107.82	0.565
**Cervicomental**	97.05	7.76	76.26	114.77	92.58	6.64	75.55	113.18	0.000***
**Medium facial third**	28.94	2.36	24.32	36.41	28.65	2.23	22.33	34.52	0.299
**Inferior facial third**	36.92	2.90	24.91	43.29	36.55	2.64	30.17	43.05	0.284
**Angle of head position**	79.28	5.39	63.01	93.51	79.46	5.34	68.57	94.13	0.785
**Facial convexity**	168.10	5.10	154.22	179.82	169.85	4.83	155.91	179.57	0.004**
**Total facial convexity**	147.03	5.14	130.89	164.21	148.83	4.58	136.96	160.00	0.003**
**Glabella-pogonion vertical**	3.59	2.63	0.00	11.81	3.93	2.39	0.00	10.37	0.277

*P<0.05; **P<0.01; ***P<0.00.

Gender differences were found in four parameters including the vertical nasal angle (N-Prn/TV), p < 0.05; Cervicomental angle (G-Pg/C-Me), p < 0.001; facial convexity angle (G-Sn-Pg), p < 0.01; and total facial convexity angle (G-Prn-Pg), p < 0.01. Larger vertical nasal angle was found in males (26.95° ± 3.69°) compared to females (25.97°± 3.67°) and a larger cervicomental angle was found in males (97.05°± 7.76°) compared to females (92.58°± 6.64°). Females exhibited larger overall facial convexity (169.85°± 4.83°) and total facial convexity angles (148.83°± 4.58°) compared to males (168.10°± 5.10°) and (147.03°± 5.14°) respectively.

## Discussion

For the most part photogrammetric studies have been conducted on small, non-random, clinical samples. This study sought to provide population norms for southern Chinese children and was obtained from a random population sample of 12-year-old children. The response rate to the study was 54.1%. The loss of participants was due to a number of factors including subjects with a history of orthodontic treatment, obstruction of landmarks and problems with photo image quality. This highlights the challenges faced in performing photogrammetric studies ‘in the field’ (in outreach settings/in non-clinical settings). Nonetheless, the sample size (of 278) was adequate to provide populations norms and to identify gender differences and is one of the largest sample size for which photogrammetric assessments have been conducted in a random population sample to date.

Norm angular values were provided for 12 angular measurements, which reflect a comprehensive range of angular assessments. Of note certain angular measurements (nasolabial & labiomental) had considerable variability. This indicates a great degree of individual variation in lip profiles amongst 12-year-olds. Similar findings in large variability of the nasolabial and labiomental angle were observed in various other studies [19,20,22,23]. Varga’s cephalometric study [22] on Croatian adolescents observed nasolabial (106.39° ± 10.36) and labiomental (130.36° ± 12.58) angles of large variability. Malkoç [20] reported large variability for the nasolabial angle (male: 75.40° to 126.90°, female: 81.71° to 129.90°) and the labiomental angle (male: 113.00° to 142.60°, female: 108.05° to 156.50°) in a sample of Class I Turkish adults. Riveiro [19] observed similar trends in variability of the nasolabial (male: 71.7° to 137.6°, female: 76.5° to 134.5°) and labiomental angles (male: 113.2° to 153.1°, female: 95.8° to 159.8°).

The mean values obtained from the present sample can be used for comparison with records of subjects evaluated using the same analysis and photogrammetric technique. Angular photogrammetic parameters can aid orthodontists and other specialists to evaluate the soft tissue aspects of the facial profile with regards to treatment planning.

In terms of method error, it was generally found to be highest for labiomental angle (2.76°), Nasolabial angle (2.50°) and Nasal angle (2.52°). This should be borne in mind in interpreting the results for clinical use. The assessment of method error is consistent to the findings of Malkoç [20] and Riveiro [19] which reported a high method error for the labiomental angle at 2.16 and 4.5 respectively. Regarding the nasolabial parameter, Malkoç [20] reported a method error of 1.60° and Riveiro [18] reported a method error of 4°. Riveiro [19] also reported a high method error for the nasal angle (3.5°).

Gender difference in 4 angular parameters were observed: vertical nasal angle, cervicomental angle, facial convexity angle and total facial convexity angle (N-Prn/TV; G-Pg/C-Me; G-Sn-Pg; G-Prn-Pg). Males had a larger vertical nasal angle (26.95°± 3.69°) than females (25.97°± 3.67° degrees). This concurs with findings from Riveiro [19] photogrammetric study on 18-20 year old Caucasian subjects. Riveiro [19] reported gender differences in the vertical nasal angle being greater in males (33.8°± 5.82°) compared to females (31.25°± 4.5°). Malkoç [20] reported no gender differences in the vertical nasal angle in a dental Class I Adult Turkish subject group between males (26.57°± 3.16°) and females (26.21°± 4.07°) with mean values comparable to the ones found in this study.

A larger cervicomental angle was found in males (97.05°± 7.76°) compared to females (92.58°± 6.64°). This concurs with the findings of Malkoç [20] with males exhibiting 104.86°± 9.86° and females 95.64°± 7.74°. Riveiro [19] also found gender differences in the cervicomental angle but reported females (84.18°± 6.65°) exhibiting a larger cervicomental angle compared to males (79.85°± 7.19°).

Females exhibited larger facial convexity (169.85°± 4.83°) and total facial convexity angles (148.83°± 4.58°) compared to males (168.10°± 5.10°) and (147.03°± 5.14°) respectively. Gender differences were not found in the study of adult subjects by Anić-Milošević [22] Malkoç [20] and Riveiro [19]. These differences may be due to variations in age groups studied or ethnic differences. Varga’s cephalometric study [23] on Class I Croatian subjects reported comparable total facial convexity angle of 141.55° in 12 – 15 year old subjects. Bergman’s longitudinal study of cephalometric soft tissue profile traits between 6 and 18 years found the facial convexity angle remained relatively constant during growth [24].

Reference values of adolescents from other ethnic groups have been compared to our findings. The nasolabial angle as a reflection of the relationship between the base of the nose and the upper lip was reported to be 99.05°± 10.24° in females and 99.03°± 11.52° in males. Farkas [25] reported similar measurements in 13 year old Canadian Whites with males: 98.6°± 9.4° and females 100.9°± 9.3°. Yuen [26] reported in 13-year-old southern Chinese the findings of 102.7°± 11.1° in males and 101.6°± 11.3° in females. Burstone reported a significantly smaller mean of 73.8°± 8.0° degrees measurement in 13 to 15 year old Caucasian children. Varga’s cephalometric study [23] on Class I Croatian children aged 12-15 years old reported a mean nasolabial angle of 106.39°.

The labiomental angle was reported to be 132.56° in males and 135.30° in females which is comparable to the findings of Yuen [26] who reported a mean of 134.3° in males and 138.3° in females in 13-year-old Southern Chinese children. Varga [23] also reported a comparable result of 130.36° in 12-15 Croatian 12-15 year-olds.

Longitudinal follow-up for this epidemiological study can be anticipated to allow further assessments of changes in soft tissue profile with growth in the Southern Chinese population.

## Conclusions

The following conclusions were drawn from the present study:

1. Norm values for the lateral soft tissue profile were presented for 12-year-old Southern Chinese population
2. High variability was found for the nasolabial, labiomental angles. These angles also exhibited high method error and should be borne in mind when interpreting results for clinical use.
3. There are gender differences in the vertical nasal, cervicomental, facial convexity and total facial convexity angles.
